# Response to the letter of Morán et al. regarding our use of an inaccurate reference for the maximal dose of vitamin C in G6PD deficiency

**DOI:** 10.1186/s13613-020-00712-5

**Published:** 2020-07-10

**Authors:** Patrick M. Honore, Herbert D. Spapen, Paul Marik, Willem Boer, Heleen Oudemans-van Straaten

**Affiliations:** 1 ICU Dept, Centre Hospitalier Universitaire Brugmann-Brugmann University Hospital, Place Van Gehuchtenplein, 4, 1020 Brussels, Belgium; 2 Medicine, Development, Ageing & Pathology Research Department, Vrije Universiteit Brussel, Brussels, Belgium; 3 Division of Pulmonary and Critical Care Medicine, Eastern Virginia Medical School, 825 Fairfax Av, Suite 410, Norfolk, VA 23507 USA; 4 Dept of Anesthesiology, Intensive Care Medicine, Emergency Medicine & Pain Medicine, Ziekenhuis Oost-Limburg, Genk, Belgium; 5 Department of Intensive Care Medicine, Amsterdam UMC, Vrije Universiteit Amsterdam, De Boelelaan 1117, 1081 HV Amsterdam, The Netherlands

To the editor,

We thank Morán et al. [1] for their very attentive reading of our review on vitamin C dosing during renal replacement therapy [2], thereby noticing that one of the cited references [3] had been retracted. We used this retracted reference to support the assertion that a vitamin C dose up to 6 g/day was not contraindicated in patients with glucose-6-phosphate dehydrogenase (G6PD) deficiency. In fact, the retraction notice appeared about 1 year after the original publication and unfortunately overlapped the first submission of our review. We sincerely apologize for missing this important item and completely agree with Morán et al. to eliminate the reference from our review.

Still, scarce data from the literature suggest that a vitamin C dose up to 6 g/day can be safely administered to patients with G6PD. Methemoglobinemia—the presence of methemoglobin in the blood—is most commonly treated with methylene blue [4]. However, methylene blue cannot be used in G6PD deficiency because it is ineffective and may even worsen G6PD deficiency-related hemolysis [4]. In vitro data going back to 1979 demonstrated that a vitamin C plasma concentration up to 5 mmol/L inhibited oxidation of oxyhemoglobin and Heinz body formation in G6PD-deficient red cells incubated with the strong oxidizing drug acetylphenylhydrazine [5]. Applying a dosing regimen of 1.5 g IV q6h, vitamin C serum concentrations are typically situated between 200 and 600 μmol/L [6]. Acute hemolytic anemia in a patient with severe methemoglobinemia and G6PD deficiency successfully resolved within 24 h following strictly monitored administration of 1 g vitamin C q6h [4]. However, a recent review reported that vitamin C doses of 4 to 6 g may propagate hemolysis [7]. This illustrates that vitamin C administration in G6PD patients requires caution. Alternative treatment [4] should be preferred if methemoglobinemia develops. In the absence of a valid alternative, a maximal IV dose of 4 to 6 g vitamin C could be considered, provided that strict monitoring is guaranteed. Meanwhile, it is wise to exclude patients with known or suspected G6PD deficiency from studies evaluating the use of 6 g vitamin C in populations that may benefit from this therapy such as septic shock or burn patients.

## Data Availability

Not applicable.

## Abbreviation

G6PD: Glucose-6-phosphate dehydrogenase
